# Aligned Chemically Etched Silver Nanowire Monolayer as Surface-Enhanced Raman Scattering Substrates

**DOI:** 10.1186/s11671-017-2358-4

**Published:** 2017-11-09

**Authors:** Jianchao Wang, Hongsheng Luo, Minghai Zhang, Xihong Zu, Zhiwei Li, Guobin Yi

**Affiliations:** Faculty of Chemical Engineering and Light Industry, Guangdong University of Technology, Guangzhou, 510006 People’s Republic of China

**Keywords:** Raman, Spectroscopy, Etching, Interfacial assembly, Alignment

## Abstract

Silver nanowires (AgNWs) were chemically etched to significantly increase the surface roughness and then self-assembled on the liquid/gas interfaces via the interfacial assembly method to obtain aligned chemically etched silver nanowire films. The as-fabricated silver nanowire films were used as novel surface-enhanced Raman scattering (SERS) substrates. The morphologies and plasmon characteristics of the substrates were investigated using multiple measurement methods. The performance of as-fabricated substrates was measured using rhodamine B as a probe. The detection limitation can be as low as 10^−11^ M. The greatly improved plasmonic properties are attributed to the efficient light coupling and larger electromagnetic field enhancement. The novel set of SERS substrates of aligned chemically etched AgNWs is believed to be important for efficient, homogeneous, and ultrasensitive SERS sensing applications.

## Background

Surface-enhanced Raman scattering (SERS) has received much attention over years as a sensitive, rapid, and noninvasive analytical technique in detection of molecules [1–3]. The Raman signal of the molecule can be increased by several orders of magnitude, especially when the molecule reside in the typical size of the field enhancement area, the so-called hot spot, which usually locate nearby sharp edges, roughened surfaces, or the junction between coupled nanometer-sized objects.

Silver nanowires (AgNWs) are an ideal SERS candidate for large surface areas and high crystallinity [4, 5]. However, SERS *hot spots* are limited to the ends of the nanowires [6]. Due to the fact that SERS hot spots are confined to a small area but dominate the overall SERS intensity of the substrate, the distribution of SERS intensity in a SERS substrate was found to be inhomogeneous, thus limiting their application as reproducible and ultrasensitive sensing platforms.

Studies have shown that when two nanowires are extremely close to each other, the electromagnetic fields at the gap region between the nanoparticles are drastically increased. Many computational models have predicted that large electromagnetic (EM) fields localized at the junction between metal structures [7, 8]. Tao and Yang [9] fabricated aligned films of silver nanowires and measured Raman intensities of probe molecule. The observed dependence on polarization direction confirms the theoretical predictions that large EM fields are localized in the interstitials between adjacent nanowires. Close-packed arrays of Ag nanowires can be easily obtained by assembly methods, including Langmuir–Blodgett [10, 11], layer-by-layer assembly [12, 13], external field [14–16], liquid–liquid interface [17, 18], and so on. Combining simple, high-yield, and good orientation regularity, the method is used to tunable control the gap size of nanoparticle. When the nanoparticles were close-packed to each other, the electromagnetic fields at the gap region between the nanoparticle pair are drastically increased [19].

To further increase the number of SERS hot spots, many efforts have been focused on roughening the surface of AgNWs, including direct metal deposition, chemical etching [20], and decorating nanowire with small metallic nanoparticles onto the AgNWs [21, 22]. These methods have been proved effective in increasing active hot spots along the Ag nanowire’s longitudinal axis. Lu et al. [23] revealed a surface plasmon-mediated photochemical etching in the presence of Raman probes. The nanoscale morphology changes on the surface of AgNW can be generated, resulting in a dramatic increase of Raman scattering intensity. Goh et al. [20] have successfully produced roughened Ag nanowires using a chemical etching method. Single nanowire SERS mapping indicated that the etched nanowires exhibited a SERS enhancement factor of ~ 10^4^, while the as-synthesized Ag nanowires only showed limited SERS signals at their tips. The result testified the advantage of chemically etched nanowires, and chemically etched nanowires are more suitable than as-synthesized nanowires for SERS substrates. However, many studies focused on single-nanowire Raman scattering or roughened AgNWs out of order; there has been little literature reporting aligned surface with roughed silver nanowires as SERS substrates by far. Furthermore, when nanowires are extremely close to each other, the electromagnetic fields at the gap region between the nanoparticles are drastically increased [24]. Herein, we present an aligned chemically etched AgNW monolayer as the SERS substrate using a chemical etching and three-phase interface assembly method. The resultant substrates were used for detection of rhodamine B (RB) with astonishing sensitivity (10^−11^ M). Repeated measurements show an excellent reproducibility of the SERS substrate. The relative standard deviations in the SERS intensities are limited to only approximately 12%. The new type of substrate provided a higher performance comparing with as-synthesized AgNWs. The findings may contribute to novel and efficient design of SERS substrates.

### Materials

AgNO_3_ (99.8%, Sinopharm Chemical Reagent Co., Ltd.), rhodamine B, poly(vinylpyrrolidone) (PVP, average molecular weight 58,000), and copper(II) chloride dihydrate were purchased from Shanghai Aladdin Bio-Chem Technology Co., Ltd. Ethylene glycol (EG), perhydrol 30% solution, and concentrated 25% ammonia solution were purchased from Tianjin Yong Da Chemical Reagent Co., Ltd. All chemicals were of analytical grade and used without further purification. Milli-Q deionized water (resistivity > 18.0 MΩ·cm^−1^) was used for all preparations.

## Methods

### Synthesis of Ag Nanowires

In a typical synthesis, EG (100 mL) was added to a three-necked round-bottom flask and heated at 160 °C for 1 h. Then, 1.5 mL of 4 mM copper(II) chloride dihydrate in EG was injected into the heated EG. After 15 min, 30 mL of 0.4 M PVP solution in EG was rapidly added to the above reactor. Using a syringe pump, 30 mL of 0.2 M AgNO_3_ was injected at a rate of 1.5 mL min^−1^ under the condition of electromagnetic stirring. The reaction was allowed to continue for about 30 min until the solution turned to opaque gray, which indicated the formation of Ag nanowires. The reaction mixture was left to cool, washed successively with acetone and water twice to remove Ag nanoparticles and excess PVP and EG, and then dispersed in ethanol.

### Etching of Ag Nanowires

Ammonium hydroxide and 30% hydrogen peroxide (9/1 *v*/*v*) was selected as an etchant. Etchant solution was always freshly prepared and kept on ice, and all the etching experiments were performed in ice-water bath. A certain amount of etchant was injected into a 4.5 mL PVP aqueous solution (1 mg mL^−1^), and the volume is 200, 300, and 400 μL, respectively. Under vigorously stirring, 500 μL of AgNWs at the concentrations of 5 mg mL^−1^ was rapidly injected under vigorously stirring. The solution immediately changed color and evolved gas; the reaction ran to completion within seconds and was allowed to maintain for another 5 min.

### Fabrication of Aligned Ag Nanowire Substrates

Five milliliters of aqueous suspension of the as-synthesized or etched AgNWs was added to the liquid surface of 25 mL chloroform in a glass vessel. An interface was formed among two immiscible liquids. One milliliter of acetone was added cautiously and dropwise to the mixture. Minutes later, a sparkling mirror-like surface emerged. The ordered Ag nanowire films were then transferred onto silicon chips. The samples of aligned chemically etched silver nanowire film were labeled as S0, S1, S2, and S3 corresponding to the amount of etchant of 0, 200, 300, and 400 μL, respectively.

### Characterization

The morphologies of the samples were observed by SEM (JEOL, JSM-7001F, Japan) and AFM (JEOL JSM-7600F, Bruker). The UV–vis absorption spectra were obtained using a UV–vis spectrophotometer (UV 2450, Shimadzu). The crystal structure was characterized by X-ray diffraction (XRD) (X’Pert Powder, Holland) with Cu-Kα line (*λ* = 0.15405 nm) in the Bragg angle ranging between 30° and 90°.

### Raman Spectroscopy

SERS spectra were obtained by using laser Raman spectroscopy (HORIBA Jobin Yvon) equipped with a 1.7 mW Ar^+^ ion laser using 633 nm light as excitation radiation. Spot diameter of laser beam was nearly 1 μm. The data acquisition time was 20 s for one accumulation. RB was selected as the probe molecule. 0.02 mL of RB aqueous solution was dropped onto the 7 × 7 mm^2^ substrate and was dispersed to a circular shape area. The same sample preparation method was used for all concentrations of RB which ranged from 10^−7^ to 10^−11^ mol L^−1^. In our experiments, the dispersed circular shape area was about 65 mm^2^, which was then dried under ambient conditions before tests. Supposing RB molecules were uniformly distributed in the circular area. The number of molecules contributing in producing a Raman signal was less than 10, when the concentration of RB is 10^−11^ mol L^−1^. The reproducibility evaluation was carried out at six randomly chosen spots from three SERS substrates. The Raman band of a silicon wafer at 520 cm^−1^ was used to calibrate the spectrometer. It should be noted that the accumulation times and the laser power are the same for all the Raman spectra.

## Results and Discussion

### Fabrication and Morphology Investigations

**Fig. 1:**
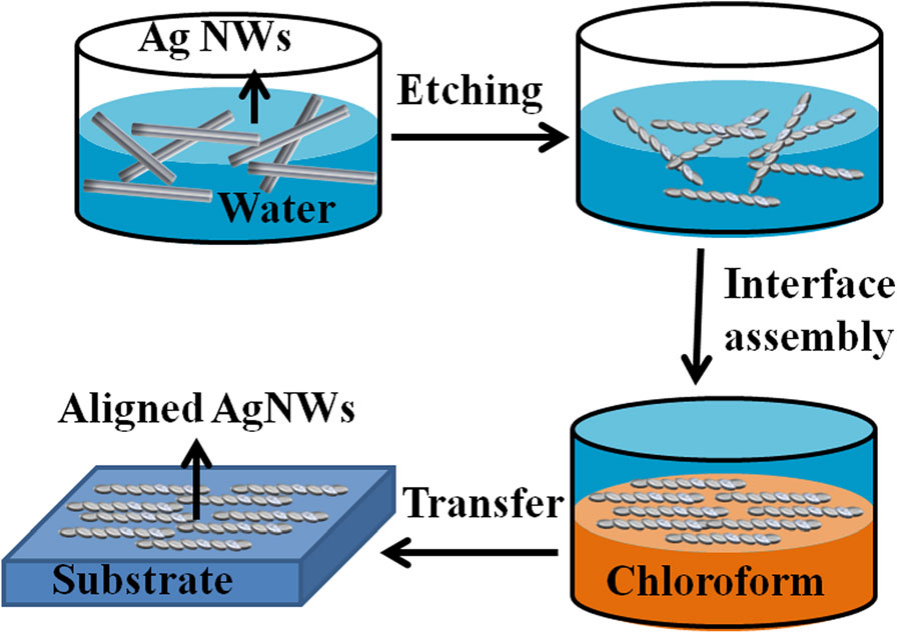
Schematic illustration of the substrate fabrication process

**Fig. 2:**
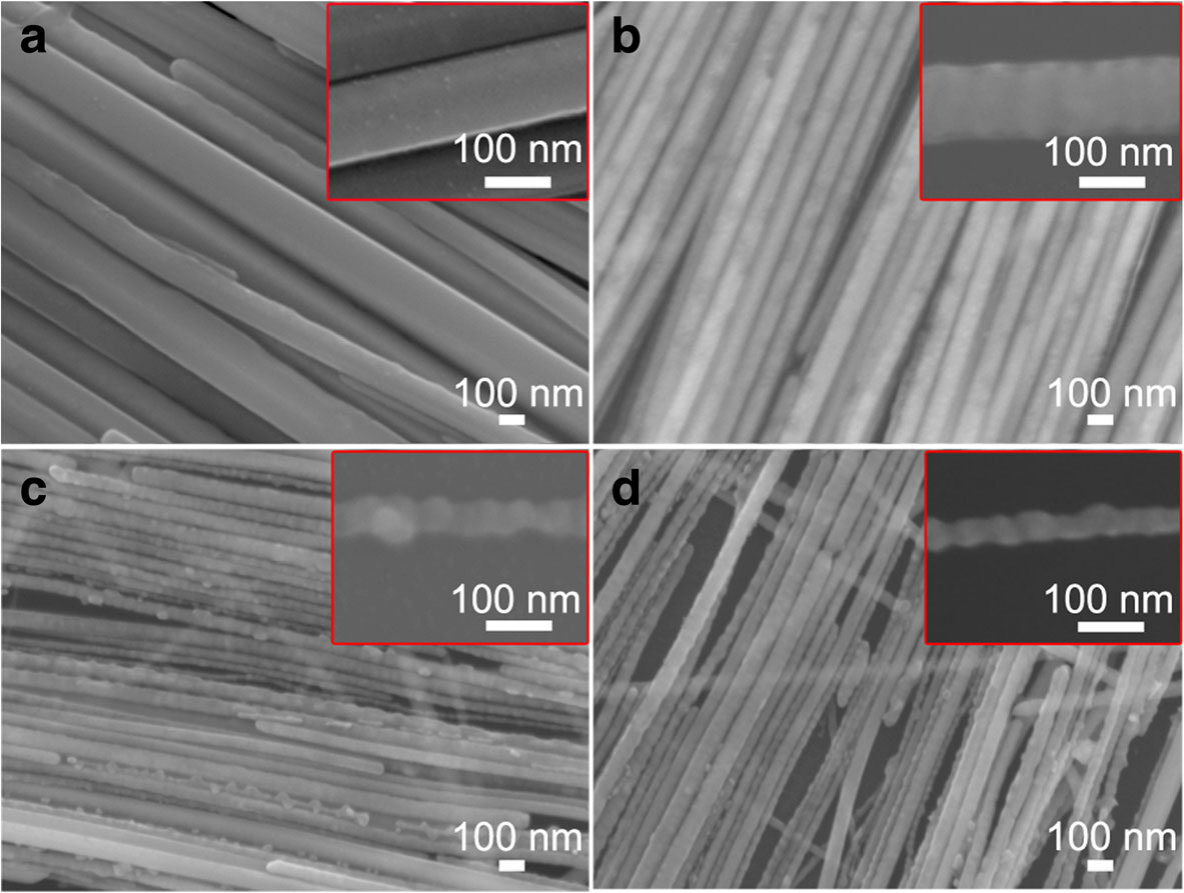
SEM images of self-assembled as-synthesized (**a**) and etched Ag nanowire film on the surface of silicon wafers using 200 μL (**b**), 300 μL (**c**), and 400 μL (**d**) 9:1 ammonia-to-hydrogen peroxide etchant solution

The fabrication process is schematically illustrated in Fig. 1, containing etching, alignment in the interface, and transferring onto the substrates. Ag nanowires were prepared by the polyol method according to the literature with little modification [25]. As-synthesized Ag nanowires show a smooth surface and uniform diameter throughout the length of the Ag nanowire. The average length and diameter of the as-synthesized Ag nanowires were 19.5 μm and 120 nm, respectively. Figure 2a shows silver nanowires with smooth surface are aligned parallel to each other, forming a close contact, and have a highly arrayed structure. Some large intervals and multilayer structure might be caused during the transfer of films from the interface to the substrate. The surface of AgNWs in S1 (Fig. 2b) was slightly roughened, and the diameter did not change significantly. Obvious waviness appeared on the surface of AgNWs in S2 (Fig. 2c), while the diameter of etched AgNWs grows smaller, and anisotropic characteristics of AgNWs were still maintained. The curve on the surface along AgNWs became more obvious, having a similar morphology of nanoscrews [26], and the diameter and length of AgNWs were further reduced in S3 (Fig. 2d). It is suggested that the surface morphology of AgNWs is sensitive to the amounts of etchant solution.

### The Surface Roughness of Silver Nanowire

**Fig. 3:**
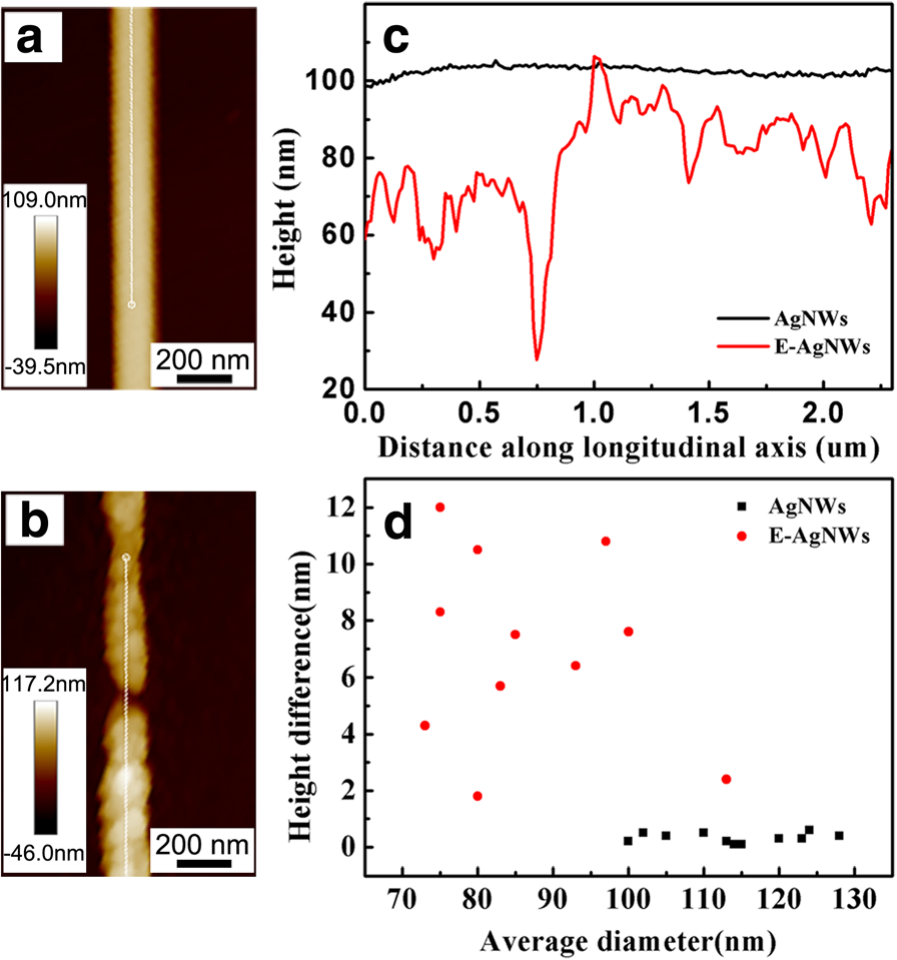
AFM height images of as-synthesized Ag nanowires (denoted as AgNWs in figures) (**a**) and chemically etched Ag nanowires (denoted as E-AgNWs, the amount of etchant was 300 μL) (**b**), AFM cross-sectional height profiles (**c**), and plot of average diameter versus height difference of as-synthesized and chemically etched Ag nanowires (**d**)

Atomic force microscopy was adopted to investigate the changes of surface roughness of silver nanowires before and after etching. Figure 3 shows representative AFM images of both as-synthesized and etched Ag nanowires. Morphology differences between two types of nanowires were obvious. The surface of as-synthesized nanowire (Fig. 3a) is smooth while the surface of etched AgNWs (Fig. 3b) became roughened and large fluctuation in height appeared. This is consistent with the observation from SEM. Figure 3c shows the height profile of both types of nanowires mentioned above. The diameter of as-synthesized nanowire appeared to be consistently about 102 nm with a variation in heights within 0.5 nm. For the etched AgNW, the average diameter was reduced to nearly 79 nm and with an approximate 10.8 nm height difference. Etching had removed a large number of silver atoms from the initial AgNWs, resulting in a considerable decrease in radius and increased roughness. Since there were diameter variations among the polyol-synthesized silver nanowires, multiple nanowires were measured to get an average result. The average diameter of a single silver nanowire versus height difference is plotted in Fig. 2d. The average diameter of as-synthesized nanowires was 114 nm, whereas for etched nanowires, the average diameter was 84 nm. These statistics clearly showed the diameter reduction upon the chemical etching process. From the *y*-axis, it is clear that the height difference between the two types of nanowires increased from 0.3 to 6.8 nm. The height difference of as-synthesized nanowires was almost negligible, while for etched nanowires, height difference is larger. The summary of diameters and height differences of multiple nanowires of both types of nanowires indicated that the chemical etching process contributed to significant changes in the diameter and surface roughness of the Ag nanowires.

**Fig. 4:**
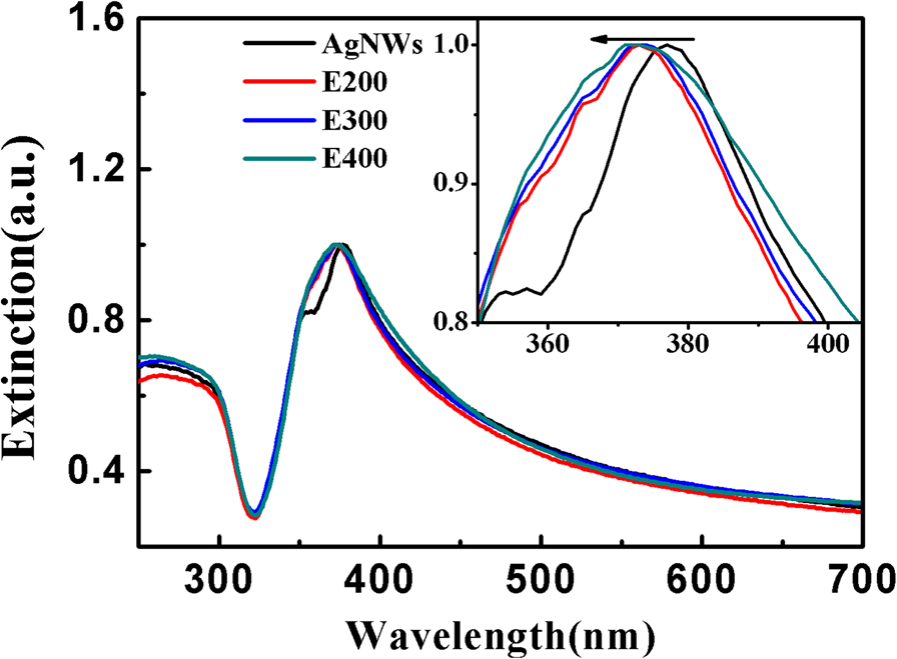
Normalized UV–vis extinction spectra for the as-synthesized and chemically etched Ag nanowire aqueous solution with different amounts of the etching agent. E200, E300, and E400 stand for the chemically etched Ag nanowires etched by 200, 300, and 400 μL etchant, respectively

Surface plasmon resonance (SPR) properties are very sensitive to modification in shapes and sizes. The SPR properties were also characterized via the UV–vis spectrum (Fig. 4). For as-synthesized AgNWs, two significant plasmon peaks were observed at 377 and 351 nm and they corresponded to the transverse plasmon resonance and quadrupole resonance excitation of nanowires, respectively [27]. However, for etched AgNWs, only one broad surface plasmon peak was observed at ~ 370 nm. The peaks near 350 nm gradually disappeared, and peaks of transverse plasmon resonance of nanowires showed slightly shifted downwards from 377 to 370 nm. The full width at half maximum became larger with the increase of etchant. This fact could be attributed to the increased surface roughness and the decrease in diameter of AgNWs.

### Crystallinity

**Fig. 5:**
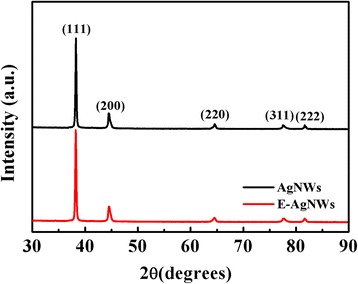
XRD pattern of the as-synthesized and chemically etched Ag nanowires

Figure 5 shows the bulk crystallinity properties of both as-synthesized and etched AgNWs by XRD spectroscopy. Both XRD patterns have five distinct diffraction peaks at 38.15°, 44.60°, 64.41°, 77.71°, and 81.58°, corresponding to (111), (200), (220), (311), and (222) crystalline planes, respectively. The positions of five diffraction peaks were very consistent with each other, and they coincided with the characteristic peaks for the fcc structure of silver according to JCPDS card file no. 4-783. Diffraction peaks of etched AgNWs have no conspicuous change, indicating that the fcc structure was preserved after chemical etching.

### Evaluation of Substrates

**Fig. 6:**
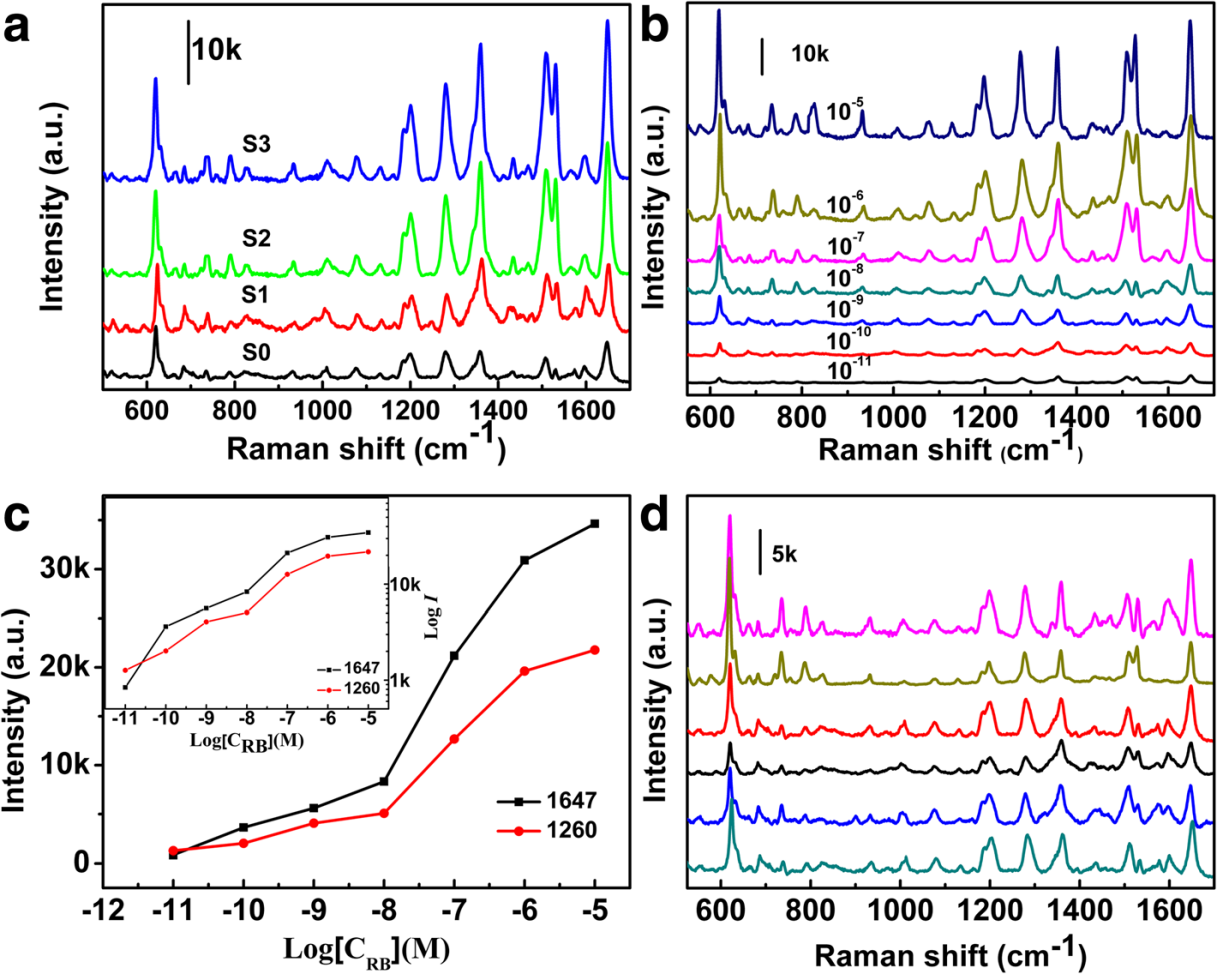
**a** SERS spectrum of RB with different substrates. RB (10^−7^ M). **b** SERS spectrum of RB with a gradient concentration. **c** The relationship between the Raman intensity and logarithmic RB concentrations for the bands at 1647 and 1260 cm^−1^. Log-log plot of the Raman intensity versus the concentration of RB is shown in the inset. **d** Six SERS spectra collected randomly in the scanned area on the substrate. RB (10^−9^ M)

SERS measurements were carried out to compare the SERS intensities of the self-assembled monolayer substrates of as-synthesized and roughened Ag nanowires using RB as the probe molecule. The corresponding spectra are collected in Fig. 6a. When the concentration of RB solution is 10^−7^ M, the peak positions of the Raman spectra were the same with different substrates. Raman bands were observed at 920, 1110, 1210, 1260, and 1330 cm^−1^, which were related to C-H stretch, C-H stretch, C-H in-plane bend, aromatic C-C stretch, and aromatic C-C stretch, respectively. It can be seen that the peak positions maintained constant upon different substrates. Whereas the Raman signals from etched silver nanowire monolayer are stronger than those from as-synthesized silver nanowire monolayer substrate, the peak intensities are gradually enhanced with the increase of etchant. The Raman enhancements were consistent with the enhancements in roughness of the surfaces, which suggested that the chemical etching method introduced a large amount of hot spot and led to a better SERS performance. We attribute such increase of Raman hot spots to the alteration of surface morphology of the nanowires. The undulating wavy ridges on the surface of etched AgNWs may act as antennas for light, where free-radiation field is localized at the ridges. As a result, more efficient light coupling and larger electromagnetic field enhancement are achieved. Such a unique feature has given rise to an increase in collective Raman scattering hot spots, offering higher SERS sensitivity. This result is consistent with the literature report [28, 29]. With highly roughened surfaces, our study reveals chemically etched nanowires are more suitable than as-synthesized nanowires for SERS substrates. Furthermore, periodic structures of arrayed silver nanowires with a close contact, parallel to each other might also provide hot spot [30], which is necessary for intense SERS enhancement.

Aligned substrates of etched Ag nanowires have been investigated with a gradient concentration (Fig. 6b) to determine the quantitative performance of the substrate and detection limit. Figure 6b shows a series of SERS spectra of RB with decreasing concentrations ranging from 1 × 10^−5^ to 1 × 10^−11^ M. The Raman spectral intensities became gradually weakened by diluting the concentrations of the RB probe molecule. The characteristic Raman peaks can still be identified until the probe concentration is reduced to a concentration of 1 × 10^−11^ M, so the detection limit of RB can reach 1 × 10^−11^ M, demonstrating the high sensitivity of this self-assembled substrates.

Figure 6c is the dependence of the peak intensity of 1647 and 1260 cm^−1^ band on logarithmic RB concentrations, in which the background of the signal has been removed in drawing the graph. The Raman intensity increased with logarithmic concentration. In the inset of Fig. 6c, a log-log plot of *I*_SERS_ versus *c*_RB_ revealed a nearly linear relationship, at low concentrations below 10^−8^ M. Above this concentration, the SERS intensity reached a plateau. This fact could be attributed to the fact that SERS intensity is proportional to the surface coverage of adsorbed molecules on hot spots, and the surface coverage follows the Hill equation [31]. The adsorption of RB became saturated beyond this level. As a result, the substrate can only be used as a reliable platform for quantitative analysis of RB at low concentrations.

In addition to high sensitivity, reproducible SERS signals are another significant issue. To assess the reproducibility of Raman signals from self-assembled substrates, SERS spectra of RB (10^−9^ M) obtained from six randomly selected positions were measured. Obviously, the SERS signals were all of comparable intensity, indicating the substrates provided uniform SERS enhancements upon its entire surface. Furthermore, we compared the intensities of the characteristic 1647 cm^−1^ line of RB in these six spectra (Fig. 6d), and the signal variations were less than 12%, far less than the internationally recognized standard (20%), suggesting the outstanding reproducibility of SERS substrate. The variations in the SERS intensities of 1647 cm^−1^ band may be attributed to differences in nanowire diameters, the distance between Ag nanowires, and the adsorption of analyte on the Ag nanowires.

**Fig. 7:**
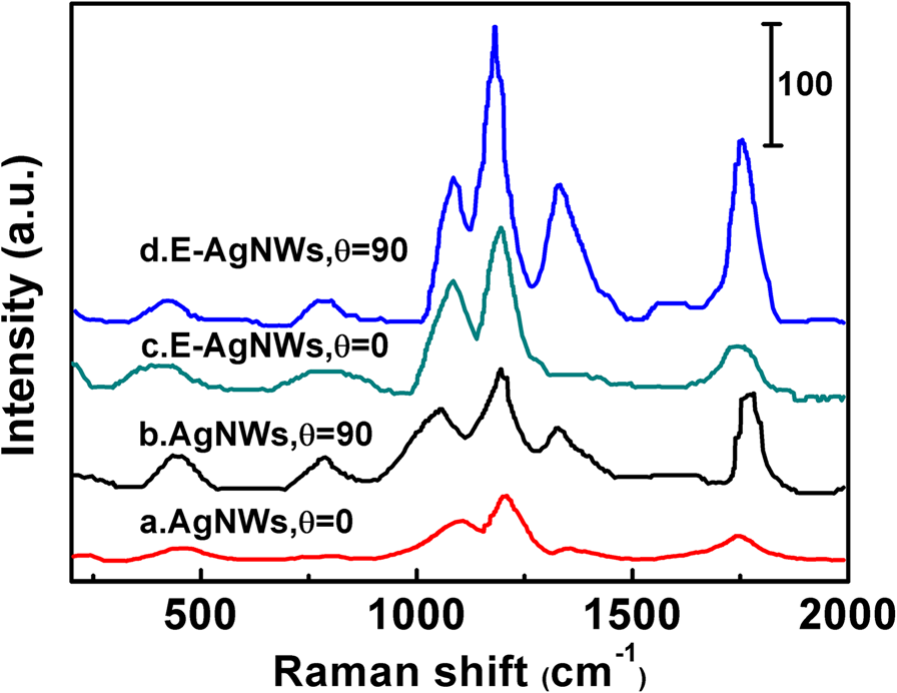
SERS spectra of RB on different nanowire monolayers taken at different polarization directions of incident light. The angle *θ* is the angle between the polarization direction and the long nanowire axis

To further demonstrate the origination of Raman enhancement, polarization dependency analysis was carried out. Figure 7 shows the Raman spectra of RB on aligned as-synthesized and etched silver nanowire substrates using polarized light parallel or perpendicular to the nanowire direction. It can be seen that for the as-synthesized silver nanowires, Raman signals generated by parallel-polarized light excitation are stronger than that those generated by perpendicular-polarized laser excitation, suggesting that the aligned silver nanowires with small interwire distance contributed to Raman enhancement. Similar results were obtained for the etched nanowire monolayers. Besides, the Raman intensity of different substrates using the same polarized light is shown in the spectra a–d (Fig. 7), and using the parallel-polarized light, the Raman intensity of etched AgNWs is stronger than that of as-synthesized AgNWs due to the surface roughening. The spots of high curvature by roughening caused intense local electromagnetic fields for *lightning rod effect* [32]. Furthermore, the intensity caused by surface roughening was found to be higher than the enhancement originated from nanowire-nanowire arrangement. The result indicated the SERS enhancement was related to nanowire arrangement and surface roughening, and the latter was dominant.

## Conclusion

Self-assembled chemically etched AgNW substrate was fabricated. The roughened surface of AgNWs was observed to increase SERS-active hot spots along its longitudinal axis, while crystallinity was preserved after etching reactions. The aligned chemically etched silver nanowire substrate has overcome the limitation of conventional one-dimensional AgNWs with limited SERS-active area serving as a platform for efficient, homogeneous, and ultrasensitive SERS sensing applications. Using RB as the probe molecule, the detection limit was 10^−11^ M. More importantly, the regularity of silver arrangement can improve the reproducibility of SERS substrate. Taking advantage of the unique generous and uniform hot spots on the surface of chemically etched Ag nanowires, we optimized the distribution of hot spots on the present substrate, thereby achieving further enhanced SERS intensity and sensitivity. This work provides a new platform for efficient, homogeneous, and ultrasensitive SERS sensing applications.
